# Redo surgery outcomes for obstructed replaced valve: a single-center experience

**DOI:** 10.1186/s43044-025-00627-1

**Published:** 2025-03-18

**Authors:** Majed Tolah, Ibraheem H. Alharbi, Hasan I. Sandogji, Ayman Abdelrehim, Nouf Lami, Thikra Alkhalaf, Albaraa Fallatah, Shyelene Utuanis, Ahmed Shabaan

**Affiliations:** 1 Madina Cardiac Center, Madinah, Saudi Arabia; 2 Ministry of Health, Madinah, Saudi Arabia; 3 Menoufia University, Shibīn Al Kawm, Egypt; 4 Taibah University, Medina, Saudi Arabia; 5 Suez Canal University, Ismalia, Egypt

**Keywords:** Redo surgery, Valves, Obstructive prosthesis, Outcomes

## Abstract

**Background:**

Obstructive prosthetic valve thrombosis is a life-threatening complication associated with high morbidity and mortality. Evaluation of outcomes of surgical management and identification of the perioperative variables associated with poor prognosis are necessary to provide appropriate interventions.

**Results:**

We conducted a retrospective analysis of 39 patients who underwent redo surgery for obstructive prosthetic valve at the Madinah Cardiac Center, Saudi Arabia. Between January 2017 to October 2023 Preoperative, intraoperative, and postoperative factors that influenced the outcome were analyzed. The nature of the obstructed valve was commonly mechanical (32/39, 82.1%) located in the mitral position (30/39, 76.9%) which occurred due to thrombosis, and the size of the thrombus was more than 1 cm in 27 (69.2%) patients. High percentage (25/39, 64.1%) of the patients had a suboptimal INR (less than 2). The major postoperative complications were respiratory failure (6/39, 15.4%) and dysrhythmias (20/39, 51.3%). The 30-day postoperative mortality was 7.7% (3/39). Patients who underwent surgery after failed thrombolysis had significantly higher mortality than those who underwent direct surgery (*p* = 0.018).

**Conclusion:**

Prosthetic valve thrombosis is primarily associated with suboptimal anticoagulant therapy and can occur years after valve replacement surgery. The prognosis for redo valve replacement is favorable with a 30-day operative mortality rate of 7.7%. For patients with prosthetic valve thrombosis, direct surgical intervention without prior fibrinolysis may be safe and effective for patients with prosthetic valve thrombosis.

## Background

Advanced rheumatic heart diseases pose a substantial hazard to public health in developing countries and are usually managed by surgical valve replacement [1]. Nevertheless, there are several risks are associated with prosthetic valves, such as biomechanical failure, endocarditis, problems related to anticoagulation and valve thrombosis, or occlusion of the prosthetic valve [2, 3].

Prosthetic valve obstruction is a serious life-threatening complication associated with high morbidity and mortality. Thrombus formation, pannus, or vegetation could be the cause of a stuck prosthesis [4]. Any obstruction of a prosthesis by noninfective thrombotic material or valve-related clotting that impairs valve function is referred to as prosthetic valve thrombosis [5]. Although the precise incidence of valve thrombosis is unknown, some authors have estimated that it can vary up to 20% in the tricuspid position and from 0.5% to 6% per patient per year in the aortic and mitral positions [5–7].

When compared to the aortic location, the incidence of prosthetic valve thrombosis is higher in mitral (5 times) and mechanical valves. The valve design is also implicated where tilting disk and ball and cage type of mechanical valve are more frequently complicated [7–9].

The effectiveness and adherence to anticoagulation and other circumstances may impact the likelihood of thrombolytic therapy or surgical intervention. The optimal treatment is still controversial; however, surgery is still the first choice for patients with NYHA functional class III or IV and not at high surgical risk [10, 11].

Identification of the perioperative variables associated with poor outcomes is necessary to inform surgical decision-making and to offer patients the most appropriate interventions [12].

This single-center study aimed to evaluate the clinical characteristics and outcomes of patients who underwent redo valve replacement surgery for prosthetic valve obstruction, focusing on identifying the perioperative factors associated with increased mortality risk.

## Methods

### Study design and settings

This single-center retrospective cohort study was conducted at the Madinah Cardiac Center (MCC), tertiary cardiac care hospital serving Madinah and the surrounding regions of Saudi Arabia.

### Ethical considerations

This study was approved by the Institutional Review Board of General Directorate of Health Affairs in Madinah (approval code: 24–072). All data were anonymized and stored securely to ensure that no identifiable patient information was accessible. This study was conducted in accordance with the ethical standards of the Declaration of Helsinki.

### Eligibility criteria

This study included patients who developed prosthetic valve obstruction and who were treated at the MCC between January 2017 and October 2023. Patients diagnosed with prosthetic valve obstruction after transcatheter aortic valve implantation (TAVI) were excluded.

We defined a prosthetic valve as a mechanical or tissue prosthetic valve implanted for patients with valvular diseases, and the patients with prosthetic stuck valve as a prosthetic valve patient with acute onset of hemodynamic instability, echocardiography showing elevated trans-prosthetic valve pressure gradient, central regurgitation, immobility or reduced leaflet mobility, and the presence of thrombus on either side of the prosthesis, with valvular obstruction.

### Data collection

All eligible patients diagnosed with an obstructed prosthetic valves during the study period were included. Data were collected by reviewing medical records. Demographic characteristics (age and sex), clinical profiles (comorbidities, underlying valve lesion, type of implanted prosthetic valve, time of implantation, anticoagulant warfarin dose, presenting symptom, echocardiographic findings before and after the redo surgery for stuck valve and thrombolytic treatment), and patient outcomes (30-day operative mortality defined as mortality due to any cause within 30 days of surgical intervention for the obstructed valve, length of stay in the ICU and hospital, duration of mechanical ventilation, incidence of postoperative respiratory failure or dysrhythmias) were collected. Survivors were followed up with serial echocardiography, which includes left ventricular and valvular functions estimation, and 19 and 13 patients completed follow-up for 2 years and 5 years, respectively.

### Patient management protocol for a prosthetic stuck valve

All patients presenting with recent changes in clinical symptoms such as dyspnea, history of palpitations, or signs of systemic embolism, were suspected of prosthetic valve obstruction. Suspected patients underwent clinical examination for peripheral arterial pulse and rhythm, cardiac auscultation for changes in murmur and valve click, and chest for the presence of crepitations. Then, transthoracic echocardiography (TTE) was performed to assess mobility and the mean gradient across the stuck valve. TTE patients were evaluated for the gradient across the prosthetic valve, the presence of thrombus or pannus which can be identified from the ultrasound intensity of mass, leaflet mobility of prosthetic valve, size of thrombus and its extension, and presence of the left atrial clot. Once the patient was diagnosed with a stuck thrombotic valve, laboratory tests such as complete blood count (CBC), coagulation profile, liver and renal function tests, ESR, CK-MB, and LDH were done. The patient was admitted to the intensive care unit (ICU) for monitoring. Patients were assessed for an increased risk of bleeding (such as low platelet count, the presence of bleeding diathesis, and INR value above the target range).

After the initial treatment of some of these patients with thrombolysis. Then, a redo valve replacement was planned. Thrombolysis was performed using streptokinase with a loading dose of 250,000 IU over one hour, followed by a maintenance dose of 100,000 IU/h via infusion. If the thrombolytic agent was not available or there was partial obstruction of the valve with mild symptoms, then heparin infusion was given with a target partial PTT 1.5–2 × of the upper normal for the patient. Otherwise, patients with obstructed prosthetic valves underwent direct redo valve replacement surgery.

### Statistical analysis

All data were tabulated and analyzed by the statistical package for the social sciences software program, IBM SPSS Statistics for Windows, version 27 (IBM Corp., Armonk, N.Y., USA). Descriptive statistics included categorical data that were presented as numbers and percentages and numerical data that were first checked for normality by the Shapiro–Wilk test. Normally distributed data were reported as the mean ± standard deviation, while not normally distributed data were presented as the median and interquartile range (25th–75th percentiles). To identify factors associated with 30-day operative mortality, associations between two categorical variables were executed by the Fishers’ exact or Fisher–Freeman–Halton exact tests. For comparison of normally distributed numerical data, we performed the independent samples T test, with the choice of equal variances not assumed for reporting of the *p*-value because of the unequal sample sizes of the two groups. Instead, the nonparametric Mann–Whitney U test was applied for comparison of the skewed numerical data. *P* < 0.05 was considered statistically significant.

## Results

This study included 39 patients who underwent redo valve replacement of obstructive prosthetic heart valves. Approximately two-thirds (*n* = 26, 66.7%) of the patients were females, and their means ages and BMI were 45.2 ± 15.9 years and 26.8 ± 6.09 kg/m^2^, respectively. Most patients (*n* = 32, 82.1%) had a history of rheumatic heart disease. Recorded comorbidities included diabetes mellitus (*n* = 11, 28.2%), hypertension (*n* = 10, 25.6%), dyslipidemia (*n* = 5, 12.8%). The patients gave a history of valve replacement surgery for the mitral (30, 76.9%), aortic (*n* = 17, 43.6%), or tricuspid (2, 5.1%) valves, and the prosthetic valve was commonly mechanical (*n* = 32, 82.1%). Most (*n* = 32, 82.1%) patients underwent one surgery. The median time between the stuck valve diagnosis and the previous valve surgery was 8.5 (IQR: 4.0–11.0) years. At presentation, there was acute pulmonary edema (15, 38.5%), dysrhythmias (*n* = 13, 33.3%), and cardiogenic shock (*n* = 13, 33.3%). Furthermore, endocarditis was detected in five (12.8%) patients. The New York Heart Association (NYHA) class III of heart failure was the most frequent (38.5%). Laboratory investigations showed elevated creatinine levels in all (100%). The initial INR was less than 2 in 25(64.1%).

Regarding transthoracic echocardiography findings, the mitral valve was in place in 27 (96.2%), while 10 (25.6%) showed an aortic valve in place. In 27 (69.2%), there was one leaflet not moving, while 12 (30.8%) showed an absence of movement in the two leaflets. Also, 12 (30.8%) showed left atrial thrombus, and the size of the occluding thrombus was more than 1 cm in 27 (69.2%). A paravalvular leak, mitral, aortic, and tricuspid regurgitation were observed (12.8%, 15.4%, 10.3%, and 17.9%, respectively). The mean LVEF% was 48.1 ± 8.9, and the means of mitral valve peak and mean gradients were 17.9 ± 6.1 and 10.0 ± 4.0, respectively, while these measures for the aortic valve were 86.7 ± 16.1 and 50.4 ± 17.6, respectively **(**Table 1**).**

**Table 1:** Demographic, clinical, laboratory, and preoperative echocardiography characteristics of the prosthetic stuck valve patients (*N* = 39)

Age, years, Mean ± SD	45.2 ± 15.9
BMI, kg/m^2^, Mean ± SD	26.8 ± 6.09
Female	26 (66.7%)
Male	13 (33.3%)
Rheumatic heart disease	32 (82.1%)
Diabetes mellitus	11 (28.2%)
Hypertension	10 (25.6%)
Dyslipidemia	5 (12.8%)
Coronary artery disease	2 (5.1%)
DVT, Pulmonary Embolism	5 (12.8%)
Cerebrovascular accident	3 (7.7%)
Mitral valve replacement	30 (76.9%)
Aortic valve replacement	17 (43.6%)
Tricuspid valve replacement	2 (5.1%)
Mechanical valve	32 (82.1%)
Tissue valve	7 (17.9%)
One surgery	32 (82.1%)
Two surgeries	6 (15.4%)
Three surgeries	1 (2.6%)
The time between the onset of manifestations and the previous valve surgery, years, Median (IQR)	8.5 (4.0–11.0)
Acute pulmonary edema	15 (38.5%)
Dysrhythmias	13 (33.3%)
Cardiogenic shock	13 (33.3%)
Endocarditis	5 (12.8%)
NYHA 0	1 (2.6%)
NYHA I	3 (7.7%)
NYHA II	10 (25.6%)
NYHA III	15 (38.5%)
NYHA IV	10 (25.6%)
Elevated serum creatinine levels (mg/dL)	39 (100%)
Elevated liver enzymes (ALT, U/L)	7 (17.9%)
Elevated lactate dehydrogenase (U/L)	25 (64.1%)
INR less than 2	25 (64.1%)
Hemoglobin less than 12 (g/dL)	21 (53.8%)
Left Atrial thrombus	12 (30.8%)
One leaflet not moving	27 (69.2%)
Two leaflets not moving	12 (30.8%)
Size of thrombus > 1 cm	27 (69.2%)
Paravalvular leak	5 (12.8%)
Mitral regurgitation	6 (15.4%)
Aortic regurgitation	4 (10.3%)
LVEF% (stuck), Mean ± SD	48.1 ± 8.9
Mitral valve peak gradient, Mean ± SD	17.9 ± 6.1
Mitral valve mean gradient, Mean ± SD	10.0 ± 4.0
Aortic valve peak gradient, Mean ± SD	86.7 ± 16.1
Aortic valve mean gradient, Mean ± SD	50.4 ± 17.6

BMI: body mass index, DVT: deep vein thrombosis, INR: international normalization ratio, LVEF: left ventricular ejection fraction, NYHA: New York Heart Association, SD: standard deviation, IQR: interquartile range

Regarding patient management, 11 (28.2%) patients received thrombolytic therapy before the redo surgical intervention. As well, Heparin was administered to 19 (48.7%) patients. In 24 (61.5%) patients, surgery was considered a high emergency and performed in less than 12 h. Redo valve replacement surgery involved the mitral valve (30, 76.9%), aortic valve (12, 30.8%), and less commonly the tricuspid valve (5, 12.8%). The new prosthetic valve was mechanical in 29 patients (74.4%) and tissue in (10, 25.6%) patients. Three (7.7%) patients needed ECMO, 1 (2.6%) needed IABP, and 8 (20.5%) were open sternum. The mean CPB time was 171.4 ± 68.7 min, while the median cross-clamp time was 103.0 (IQR: 86.0–140.0) minutes. After the operation, the LDH levels were elevated in 5 (12.8%) patients, and the mean LVEF% was 47.8 ± 9.2. The means of mitral valve peak and mean gradients showed lower values than that of the preoperative measures (7.6 ± 2.6 and 3.3 ± 1.1, respectively). The measures for the aortic valve also showed an improvement (22.6 ± 7.9 and 12.7 ± 4.8, respectively). There was moderate pulmonary hypertension was observed in 18 (46.2%) patients, while severe pulmonary hypertension was observed in two (5.1%). The mean LVEF% at discharge was 46.5 ± 10.0, and follow-up revealed the final LVEF% of 47.1 ± 11.4 **(**Table 2**).**

**Table 2:** Thrombolytic therapy, operative, and postoperative echocardiography characteristics of the prosthetic stuck valve patients (*N* = 39)

Preoperative thrombolytic therapy	11 (28.2%)
Preoperative heparin therapy	19 (48.7%)
High emergency surgical intervention in less than 12 h	24 (61.5%)
Operation on MV	30 (76.9%)
Operation on AV	12 (30.8%)
Operation on TV	5 (12.8%)
New mechanical valve	29 (74.4%)
New tissue valve	10 (25.6%)
ECMO machine	3 (7.7%)
IAPB	1 (2.6%)
Open sternum	8 (20.5%)
CPB time, minutes, mean ± SD	171.4 ± 68.7
Cross clamp time, minutes, median (IQR)	103.0 (86.0–140.0)
Elevated serum creatinine levels (mg/dL)	39 (100%)
Elevated lactate dehydrogenase (U/L)	5 (12.8%)
Postoperative LVEF%, mean ± SD	47.8 ± 9.2
MV peak gradient, mean ± SD	7.6 ± 2.6
MV mean pressure gradient, mean ± SD	3.3 ± 1.1
AV peak gradient, mean ± SD	22.6 ± 7.9
AV mean pressure gradient, mean ± SD	12.7 ± 4.8
TV peak gradient, mean ± SD	27.9 ± 12.1
Mild pulmonary hypertension, N %	3 (7.7%)
Moderate pulmonary hypertension. N %	18 (46.2%)
Severe pulmonary hypertension, N %	2 (5.1%)
Discharge LVEF%, mean ± SD	46.5 ± 10.0
Last LVEF%, mean ± SD	47.1 ± 11.4

AV: aortic valve, CPB: cardiopulmonary bypass, ECMO: extracorporeal membrane oxygenation, IAPB: intra-aortic balloon pump, IQR: interquartile range, LVEF: left ventricular ejection fraction, MV: mitral valve, NYHA: New York Hear Association, SD: standard deviation, TV: tricuspid valve

Table 3 shows the outcomes of surgical replacement of obstructive prosthetic valves. Six (15.4%) patients developed postoperative respiratory failure and (n = 20, 51.3%) developed dysrhythmias. The median duration of mechanical ventilation was 3.0 (IQR: 2.0- 5.0) days, and the length of ICU stay was 7.0 (IQR: 5.0- 8.0) days. The incidence of death within 30 days of surgery was 7.7% (three patients), and the mechanism of death was cardiac arrest.

**Table 3:** Outcomes of the surgical management of prosthetic stuck valve patients

Postoperative respiratory failure	6 (15.4%)
Postoperative dysrhythmias	20 (51.3%)
Thirty-day operative mortality	3 (7.7%)
Cause of death (cardiac arrest)	3 (7.7%)
Mechanical ventilation duration (days), median (IQR)	3.0 (2.0–5.0)
Length of ICU stay (days), median (IQR)	7.0 (5.0–8.0)
Length of hospital stay (days), median (IQR)	22.5 (15.0–27.5)

IQR: interquartile range, ICU: intensive care unit

The preoperative NYHA class showed a significant negative association with the 30-day operative mortality (*p* = 0.033). Out of the three deaths, two (66.6%) were class I and 1 (33.3%) was class III. Furthermore, mortality was significantly higher in patients who underwent surgery after failed thrombolysis (*p* = 0.018) than in those who underwent direct surgery. In addition, the median CPB time of the non-survivors (259.0 min) was significantly longer than that in survivors (141.5 min), *p* = 0.009. No significant associations were observed between 30-day operative mortality and demographics, preoperative comorbidities, clinical status including dysrhythmias and cardiogenic shock, echocardiographic findings such as the presence of left atrial thrombus, the obstructing thrombus size, number of leaflets not moving, and LVEF% (all *P* values > 0.05) **(**Table 4**).**

**Table 4:** Factors associated with 30-day operative mortality after surgical management of prosthetic stuck valve

Preoperative, operative, and postoperative variables		Mortality within 30 days of surgery				*P*-Value
		No *N* = 36		Yes *N* = 3		
Gender	Female	23	63.9%	3	100.0%	0.538
	Male	13	36.1%	0	0.0%	
Age, years	Mean ± SD	45.51 ± 6.4 26.7 ± 6.3		41.0 ± 8.2 27.5 ± 2.9		0.643
BMI, kg/m^2^	Mean ± SD					0.836
Rheumatic heart disease	Yes	29	80.6%	1.00	100.0%	1.00
Diabetes mellitus	Yes	11	30.6%	0	0.0%	0.545
Preoperative acute Pulmonary Edema	Yes	12	33.3%	3	100.0%	0.050
Preoperative dysrhythmias	Yes	12	33.3%	1	33.3%	1.00
Preoperative NYHA	0	1	2.8%	0	0.0%	0.033*
	I	1	2.8%	2	66.7%	
	II	10	27.8%	0	0.0%	
	III	14	38.9%	1	33.3%	
	IV	10	27.8%	0	0.0%	
Preoperative cardiogenic shock	Yes	12	33.3%	1	33.3%	1.00
Left Atrial thrombus	Yes	12	33.3%	0	0.0%	0.539
Number of leaflets not moving	1	26	72.2%	1	33.3%	0.219
	2	10	27.8%	2	66.7%	
Size of thrombus > 1 cm	Yes	24	66.7%	3	100.0%	0.539
Preoperative LVEF%	Mean ± SD	48.4 ± 8.9		45.0 ± 10.01		0.621
Postoperative LVEF%	Mean ± SD	48.2 ± 8.9		43.3 ± 2.5		0.575
Preoperative serum LDH	Median (IQR)	334.0(215.5–431.0)		3.30 (2.40–3.40)		0.007*
Preoperative serum CK-MB	Median (IQR)	32.5 (23.7–81.0)		34.0 (15.0–561.0)		0.785
High emergency surgical intervention	Yes	22	61.1%	2	66.7%	1.00
Thrombolytic therapy	Yes	8	22.2%	3	100.0%	0.018*
Heparin therapy	Yes	0.106		0.106		0.106
Operation on MV	No	8	22.2%	1	33.3%	0.556
	Yes	28	77.8%	2	66.7%	
Operation on AV	Yes	11	30.6%	1	33.3%	1.00
Operation on TV	Yes	4	11.1%	1	33.3%	0.345
CPB time, minutes	Median (IQR)	141.5 (120.0 210.5)		259.0(231.0–342.0)		0.009*
Cross clamp time, minutes	Median (IQR)	101 (86.5–130.5)		183.0 (49.0–186.0)		0.564

^*^Significant at *p* < 0.05

ALT: alanine aminotransferase, AV: aortic valve, BMI: body mass index, CPB: cardiopulmonary bypass, CK-MB: creatine kinase-MB, DVT: deep vein thrombosis, ECMO: extracorporeal membrane oxygenation, IQR: interquartile range, LDH: lactate dehydrogenase, LVEF: left ventricular ejection fraction, MV: mitral valve, NYHA: New York Hear Association, SD: standard deviation, TV: tricuspid valve

Thrombolysis was administered as initial management only in 11 (28.2%) patients and heparin in 19 (48.7%) patients. Surgery was the definitive therapy for all patients in this study after failure of medical management. All stuck valves were replaced with mechanical (29/39) or tissue valve (10/39). Valve replacement was performed as a high emergency in less than 12 h in 61.5% (24/39) patients.

Concerning patients outcomes, the major morbidity events after the redo surgery were respiratory failure (6/39, 15.4%) and dysrhythmias (20/39, 51.3%). The 30-day operative mortality rate was 7.7% (3/39), where in dysrhythmias and cardiac arrest caused death. Furthermore, 30-day operative mortality was significantly higher among NYHA class I patients (2/3, 66.6%). Death occurred in one NYHA class III patient and none of the NYHA class IV patients.

## Discussion

This study aimed to evaluate the features and outcomes of the patients undergoing revision valve replacement surgery for prosthetic valve obstruction and to identify the relationship between preoperative, intraoperative, and postoperative factors and early death within 30 days of surgery.

In this study, the stuck valve was removed and replaced in the presence of a thrombus which was more than 1 cm in 27 (69.2%) patients. We also detected endocarditis and paravalvular leakage in five (12.8%) patients. In comparison, the reported causes in other studies for redo valve replacement surgery of a prosthesis include obstructing thrombosis or thromboembolism, pannus formation, paravalvular leakage, and endocarditis [13–15]. Prosthetic valve endocarditis is a rare but catastrophic complication. Surgical intervention for endocarditis is necessary in patients with complications associated with valvular obstruction [16].

Patients with prosthetic mechanical valves require life-long anticoagulant therapy and should attain an optimal INR level to prevent thrombosis [17]. Our study had a high percentage (25/39, 64.1%) of patients with a suboptimal INR (less than 2) which contributed to the development of the occluding thrombus. similar results were found in another study in which 55.5% of the patients had suboptimal INR [18].

A study from Pakistan highlighted the role of poor compliance with warfarin therapy and high suboptimal INR (82.86%) as the main causes of prosthetic valve thrombosis [19].

Most case series reported by Tadesse et al. [20] also showed a suboptimal anticoagulant therapy reflected by their low INR level. Therefore, regular follow-up after the initial valve replacement and adequacy of anticoagulation therapy are of paramount importance to minimize this life-threatening condition. Furthermore, mechanical valves and mitral position have been reported as frequent risk factors for prosthetic valve thrombosis [7], which were consistent with our study results, showing mechanical valves (32, 82.1%) and mitral position (30, 76.9%) as frequent risk factors.

In the current study, the median time between valve replacement and onset of prosthetic valve obstruction was 8.5 years (IQR: 4.0- 11.0). A case series from Ethiopia reported a median follow-up of 36 months [20]. While a study from South Africa reported a median follow-up time of four years, with an increased risk of thrombosis during the first year following the surgery (24%) [21].

Although current guidelines favor thrombolysis, its strict indication and high incidence of complications including bleeding, thromboembolism, and recurrence limit its use to the presence of a small recent thrombus or in critical condition requiring surgery [22]. Hence, surgery is the definitive therapy for valve obstruction despite its high risk and mortality. A high recurrence rate limits thrombectomy, an alternative option [23].

Mortality was also significantly higher in patients who underwent surgery after thrombolysis failure than in those who underwent direct surgery. Furthermore, the non-survivors had significantly longer CPB time than survivors.

Variable mortality rate are associated with prosthetic valve thrombosis management. The degree of severity of thrombosis and the treatment modality involved influence the death rate [4]. Inamdar et al. [4] stated that surgical treatment is usually preferred as life-saving in cases of obstructive prosthetic valve thrombosis. Surgery is the mainstay modality of treatment for hemodynamically stable patients, while unstable patients should undergo thrombolysis, be stabilized, and then be operated on for valve replacement. Alternatively, Roudautet al. [24] and Renzulli et al. [25] confirmed that surgery is safer than fibrinolysis, and prompt surgical treatment is associated with a better early success rate and significantly lower incidence of complications in prosthetic heart valve obstruction. In cases of left-sided obstructive PVT, surgery is recommended for NYHA class III and IV patients unless there is a high surgical risk [26].

In this study, the mitral valve was the most commonly re-operated stuck valve (30/39, 76.9%) and the 30-day operative mortality was low among NYHA classes III and IV. These findings are inconsistent with those of Onorati et al. [27] who reported that mortality rates with redo surgery depend on the valve involved and the clinical status of the patient, with higher mortality rates associated with mitral involvement and hemodynamic instability.

A study from Pakistan [19] reported a higher incidence (16.6%) of deaths after redo surgery for patients who failed to respond to fibrinolytic therapy. The authors attributed this high death rate either to the increased patients’ risk for surgery after failure of fibrinolytic therapy, or to the operated valve type (mitral prosthesis). Another single-center study in India reported a total mortality rate of 29.2% (19/65) after redo surgery for obstructive prosthetic valve thrombosis of the mitral valve and a mean follow-up duration of 1.33 ± 1.03 years. Mortality was significantly higher among NYHA class III and IV patients, and among those who underwent direct surgery without preceding thrombolysis [15].

A recent study from Egypt reported on redo surgery in 27 patients with mechanical mitral and aortic valve obstructions. Emergency surgery was performed in all cases without preceding fibrinolysis, and included valve replacement, thrombectomy, or pannus removal. Follow-up revealed the death of two patients (7.4%), one died early due to acute heart failure, and the second patient died late as a result of endocarditis [28].

Furthermore, redo mitral valve replacement surgery for 64 patients at a tertiary South African center was associated with the death of two (3.1%) patients during the postoperative period and a postoperative complication rate of 15.6% [29].

An earlier study involved 39 patients presented with prosthetic valve thrombosis at the Montreal Heart Institute, Canada. Valve thrombectomy or replacement was done as a redo surgery. The reported overall 30-day operative mortality was 25%, and the 30-day operative mortality in patients who underwent valve replacement was 9% (3/32) [30]. Another 10-year experience of surgical management of 18 patients with prosthetic valve thrombosis by valve replacement revealed a higher 30-day mortality of 16.7% [31].

### Limitations

This study had some limitations. The retrospective nature of the study inherently carries the risk of selection and information biases, the relatively small sample size further limited the ability to draw robust conclusions, particularly regarding less common outcomes and associations. Finally, the variability in the patient follow-up, with some patients followed up for as little as 2 years, may have limited the ability to comprehensively assess long-term outcomes and complications.

## Conclusion

Prosthetic valve thrombosis is primarily associated with suboptimal anticoagulant therapy and can occur years after valve replacement surgery. The prognosis for redo valve replacement is favorable, with a 30-day operative mortality rate of 7.7%. For patients with prosthetic valve thrombosis, direct surgical intervention without prior fibrinolysis may be safe and effective for patients with prosthetic valve thrombosis.

## Data Availability

No datasets were generated or analyzed during the current study.

## Abbreviations

PVE: Prosthetic valve endocarditis
TTE: Transthoracic echocardiography
TEE: Transesophageal echocardiography
ESR: Erythrocyte sedimentation rate
INR: International normalized ratio
ICU: Intensive care unit
CK: Creatine kinase
